# Anterior Levator Muscle Resection and Switch in a Patient With Severe Blepharoptosis and Poor Bell’s Phenomenon: A Case Report

**DOI:** 10.7759/cureus.63347

**Published:** 2024-06-28

**Authors:** Fatema Aljufairi, Lee Cheuk Lam, Jake Uy Sebastian, Kenneth Lai, Kelvin Chong

**Affiliations:** 1 Department of Ophthalmology and Visual Sciences, The Chinese University of Hong Kong, Hong Kong, HKG; 2 Ophthalmology Department, Salmaniya Medical Complex, Manama, BHR; 3 Department of Ophthalmology, The University of Hong Kong, Kowloon, HKG; 4 Department of Ophthalmology, Vicente Sotto Memorial Medical Center, Cebu City, PHL

**Keywords:** novel technique, poor bell’s, levator switch, blepharoptosis, ptosis

## Abstract

This case report describes a novel surgical technique, the "levator switch," for correcting severe blepharoptosis in a 65-year-old man with poor Bell's phenomenon following previous bilateral ptosis surgery. He presented with recurrent ptosis, weak levator function, and excessive frontalis muscle use. The technique involves a sequential approach: anterior levator resection followed by repurposing the resected tissue as a posterior lamellar graft to the lower tarsal border. This elevates the eyelid margin while maintaining a stable palpebral fissure height. The levator switch addresses ptosis from poor levator function and minimizes postoperative corneal exposure. It offers advantages over the existing tarsal switch procedure by preserving the tarsus and meibomian glands, thus maintaining eyelid stability and contour.

## Introduction

Blepharoptosis, also known as ptosis, refers to the drooping of the upper eyelid in a straight-ahead gaze [1]. This condition can be caused by congenital factors present at birth or acquired later in life. Acquired ptosis can have various causes, including aponeurotic, neurogenic, myogenic, traumatic, and mechanical [2]. Myopathic ptosis can cause impaired ocular movement and weak orbicularis oculi muscle, which results in poor Bell's phenomenon that increases the risk of corneal exposure postoperatively [3].

The surgical correction of ptosis can be a challenging task, particularly in cases where the levator muscle function is suboptimal [4]. The severity of the condition requires a specialized approach tailored to the specific needs of the patient. However, while tarsal resection can improve the appearance of the eyelid, it can also lead to corneal exposure, dry eyes, and meibomian gland dysfunction [5]. Anterior levator muscle switch is a novel surgical technique used to address patients with severe blepharoptosis with co-existing poor Bell's phenomenon. We present a case of anterior levator muscle resection and switch procedure in a patient with severe blepharoptosis with a high risk of corneal exposure.

This article was presented as a meeting abstract in the World Society of Ophthalmic Plastic Reconstructive & Aesthetic Surgery (WSOPRS) Meeting 2023 and as a video in the American Academy of Ophthalmology (AAO) Annual Meeting 2023.

## Case presentation

**Figure 1:**
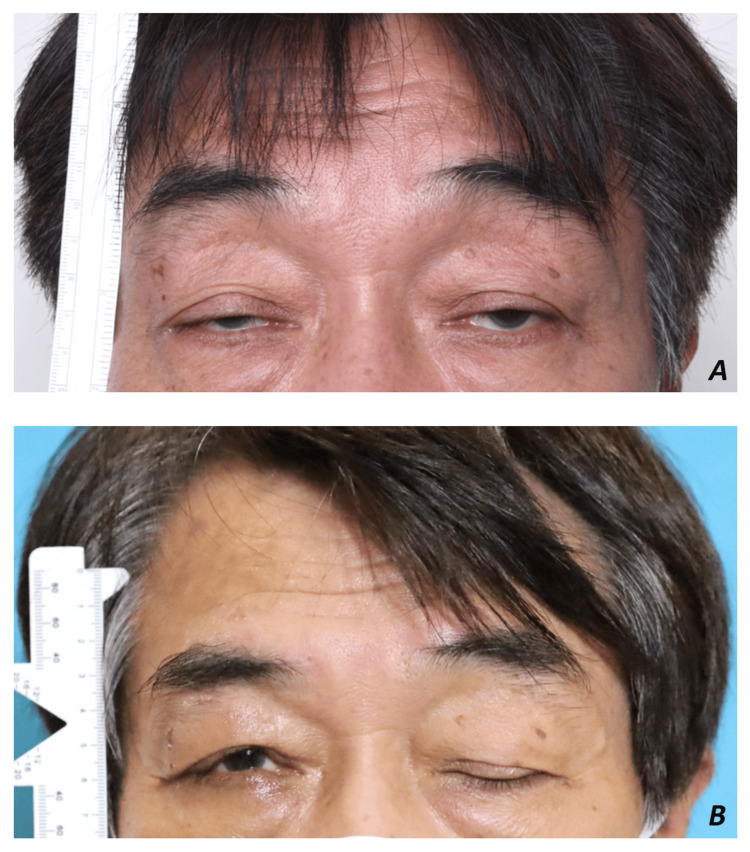
A: Right eye preoperative clinical photo. B: Right eye postoperative clinical photo.

A 65-year-old gentleman underwent bilateral muller muscle conjunctival resection, tarsectomy, and later bilateral levator resection for recurrent ptosis done elsewhere two years prior to presentation. He presented with bilateral recurrent ptosis (margin reflex distance 1 (MRD1) = 0 mm, MRD2 = 5 mm), poor levator function (5 mm), poor Bell’s phenomenon, and severe frontalis overaction (Figure 1A).

Surgical technique

**Figure 2:**
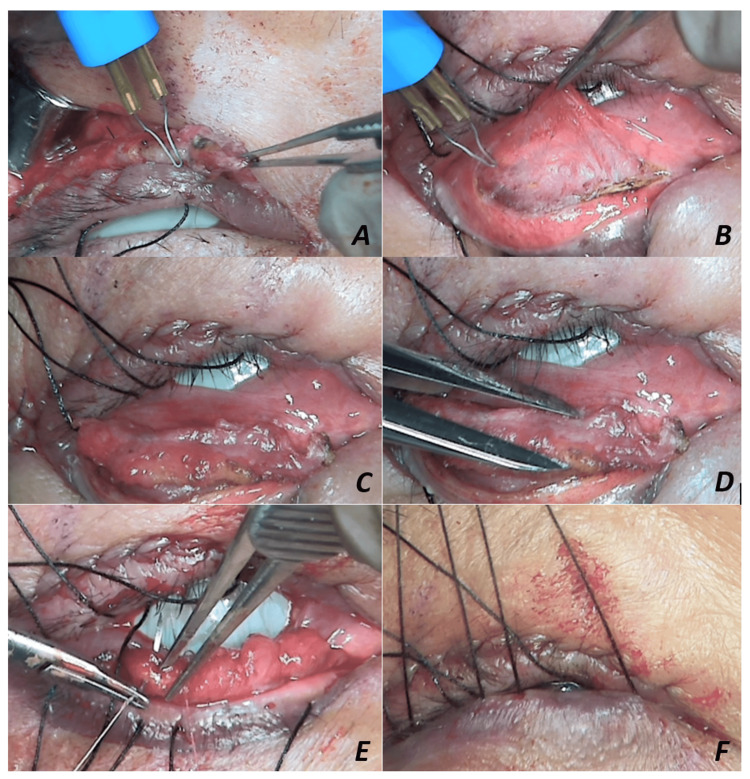
A: Redundant levator aponeurosis excision. B: Lower eyelid retractor recession. C: Repurposed aponeurosis as a lower-lid posterior lamellar graft. D: Fashioning the height of the resected levator aponeurosis. E: Suturing of aponeurosis to the inferior tarsal border. F: Frost sutures on the upward traction.

The surgical procedure was performed under local anesthesia through an eyelid crease skin incision. A sequential operative approach was adopted by anterior levator resection. The desired level of the anterior levator advancement was secured to the superior tarsal border, and intraoperative lid height and contour adjustments were done with the patient upright. The redundant levator aponeurosis (5-6 mm) was resected (Figure 2A) and transferred as a posterior lamellar spacer graft for the lower eyelid positioned between the inferior tarsal border and the recessed lower lid retractors (Figures 2B, 2C) to elevate the MRD2, thus keeping a stable palpebral fissure height (5 mm) (Figure 2D). The graft was then secured with 6-0 Vicryl (Figure 2E). Bandage contact lens and frost traction sutures were applied to the lower eyelid and placed on upward traction postoperatively (Figure 2F).

At three months postoperative, the left visual axis was unobstructed with MRD1 = 2 mm, MRD2 = 3 mm, and <1 mm lagophthalmos (Figure 1B).

## Discussion

Several methods have been used to treat ptosis, namely, Müller’s muscle conjunctival resection tarsal resection, tarsal switch, levator advancement, and frontalis slings or flaps surgery. Each technique has its own advantages and sets of drawbacks.

The correction of ptosis often leaves the cornea exposed postoperatively. The tendency to undercorrect ptosis surgery is well justified, especially in the presence of suboptimal Bell’s phenomenon and preexisting dry eye disease, which may lead to eventual ptosis recurrence. We propose elevating MRD1 and MRD2 using the resected levator aponeurosis to provide a single solution for such a situation. The resulting correction provides an upward shift of the palpebral fissure height, freeing the visual axis while maintaining a stable palpebral fissure height, which safeguards the cornea from postoperative exposure. These outcomes allow the patient to resume daily activities with less discomfort, improve aesthetics, mitigate the risk of ocular damage, and improve their overall quality of life [6,7]. A literature review on tarsal switch surgery and their respective outcomes and adverse events are outlined in Table 1.

**Table 1:** Literature review of the published articles on the surgical technique of the tarsal switch NA: not applicable

Author (year)	No. of cases	Methods	Outcomes	MRD1 (Median)	MRD2 (Median)	Palpebral fissure
DeMartelaere et al. (2006) [5]	26 eyelids (14 patients)	Posterior approach tarsal switch procedure	Thirteen patients (93%) recovered fully. Fourteen patients had palpebral fissure repositioned cephalad. No intraoperative complications. Fourteen patients were satisfied. One patient developed lagophthalmos postoperative.	NA	NA	NA
Lucci et al. (2009) [7]	9 eyelids (6 patients)	Posterior approach tarsal switch procedure	The palpebral fissure repositioned cephalad, unmasking their visual axis in the primary position, leading to improvement in their head position. No exposure symptoms postoperatively.	NA	NA	4.1 mm (range, 3 to 5 mm)
Lenake and McNab (2017) [3]	6 eyelids (9 patients)	Posterior approach tarsal switch procedure	No intraoperative complications. No significant corneal exposure. No revision required over a follow-up period of six to 52 months (mean 16 months).	Median: 4.5 mm; mean: 2.3 mm (+1.5 mm)	Median: 2.5 mm; mean: 1.6 mm (-0.5 mm)	NA
Meneghim et al. (2018) [8]	18 eyelids (11 patients)	Anterior approach tarsal switch procedure	Postoperatively: Good lid margin contour Improved chin position. Patients were satisfied with the surgical outcomes. One patient's lower lid was poorly positioned and a tarsal strip procedure was done for correction. One patient at 10 months postoperatively had a recurrence of ptosis corrected with resection of the levator aponeurosis. One patient developed punctate keratitis postoperatively, which improved with topical lubricants.	From 0.0 mm to 1.0 mm (median, +1.75 mm; mean, +2.3 mm; range, +0 to 7 mm)	From 4.5 mm to 3.0 mm (median, -1.5 mm; mean, -1.8 mm)	From 4.0 mm to 4.0 mm (mean, +0.8 mm; range, −1.5 to 7 mm)

Previous tarsal switch techniques have been reported to increase the MRD1 while decreasing the MRD2 [8], and while it remains an effective means for correcting severe ptosis, it was associated with MGD due to the tarsal plate resection. Two published works on tarsal switches reported postoperative myopathic lagophthalmos as a recurring complication [5,8].

## Conclusions

Corneal exposure and dry eye are common in patients with myopathic ptosis. The novel levator switch technique offers an effective alternative treatment to correct ptosis with both poor and normal levator muscle function, when Bell's phenomenon is absent or poor. This procedure minimizes the risk of corneal exposure and preserves the meibomian glands by sparing tarsal plate resection. However, further large-scale studies are required to definitively confirm its long-term efficacy.
